# Effects of Coffee and Tea Consumption on Glucose Metabolism: A Systematic Review and Network Meta-Analysis

**DOI:** 10.3390/nu11010048

**Published:** 2018-12-27

**Authors:** Yoshinobu Kondo, Atsushi Goto, Hisashi Noma, Hiroyasu Iso, Kunihiko Hayashi, Mitsuhiko Noda

**Affiliations:** 1 Department of Endocrinology and Metabolism, Graduate School of Medicine, Yokohama City University, 3-9 Fukuura, Kanazawa-ku, Yokohama, Kanagawa 236-0004, Japan; kondoycu@gmail.com; 2 Division of Epidemiology, Center for Public Health Sciences, National Cancer Center, 5-1-1 Tsukiji, Chuo-ku, Tokyo 104-0045, Japan; 3 Department of Data Science, Institute of Statistical Mathematics, 10-3 Midori-cho, Tachikawa, Tokyo 190-8562, Japan; noma@ism.ac.jp; 4 Public Health, Department of Social and Environmental Medicine, Osaka University Graduate School of Medicine, 2-2 Yamadaoka, Suita-shi, Osaka 565-0871, Japan; iso@pbhel.med.osaka-u.ac.jp; 5 Department of Basic Medical Sciences, School of Health Sciences, Gunma University, 3-39-15 Showa-machi, Maebashi, Gunma 371-8514, Japan; khayashi@gunma-u.ac.jp; 6 Department of Endocrinology and Diabetes, Saitama Medical University, 38 Morohongo, Moroyama-machi, Iruma-gun, Saitama 350-0495, Japan; noda_m@saitama-med.ac.jp

**Keywords:** coffee, tea, glucose metabolism, fasting blood glucose, network meta-analysis

## Abstract

Prospective cohort studies have described an association between coffee or tea consumption and the risk of developing diabetes. However, whether coffee or tea improves glucose metabolism remains uncertain. We investigated the effect of coffee and tea on glucose metabolism by conducting a systematic review and meta-analysis of randomized controlled trials. Electronic databases were searched for articles published up 19 February 2017. The primary endpoint was the mean difference in post-intervention fasting blood glucose (FBG) levels between the groups. Of 892 citations screened, 27 studies (1898 participants) were included in our meta-analysis. A network meta-analysis suggested that green tea, but not caffeinated/decaffeinated coffee or black tea, may reduce FBG levels, compared with placebo/water (−2.10 mg/dL; 95% confidence interval (CI), −3.96 to −0.24 mg/dL; *p* = 0.03; moderate quality of evidence). In a subgroup analysis, the effect of green tea on FBG levels was statistically significant only in studies with a mean age of < 55-years-old or Asian-based studies. The oolong tea group also showed a significant decrease in FBG, but the quality of evidence was very low. In conclusion, green tea consumption might decrease FBG levels, especially in < 55-year-olds or Asian-based populations.

## 1. Introduction

The number of people with diabetes is increasing rapidly, worldwide. The International Diabetes Federation reported that in 2015, 415 million adults had diabetes and that by 2040, this number is expected to rise to 642 million [1]. In 2015, the global economic burden associated with diabetes reached US$1.31 trillion, becoming a substantial global economic burden [2]. Therefore, low-cost and easily accessible strategies for preventing diabetes are required. Globally, coffee and tea are widely consumed beverages and their consumption is integrated into people’s daily lives. A large body of epidemiological evidence, from prospective cohort studies, suggests a strong association between coffee/tea consumption and a decreased risk of diabetes [3,4,5,6]. If the associations are causal, there would be substantial public health implications. However, whether or not these beverages reduce glucose metabolism remains uncertain. Previous meta-analyses of clinical trial data have investigated the effects of coffee or tea consumption on glucose metabolism [7,8,9]. Unfortunately, these meta-analyses have not evaluated tea and coffee comprehensively; have included duplicate studies [7]; have evaluated only acute effects [10]; and did not evaluate the quality of evidence across studies [7,8,9], using approaches such as the Grading of Recommendations Assessment Development and Evaluation (GRADE) system [11]. Together, these weaknesses limit the interpretation of the results and provide insufficient information to make relevant judgments.

Network meta-analysis (NMA) is a method that enables the comparison of multiple interventions using direct and indirect comparison evidence even when direct comparisons of data are insufficient [12]. Hence, we performed a systematic review that involved an NMA, using the GRADE system to comprehensively evaluate the effects of coffee and tea consumption on glucose metabolism.

## 2. Materials and Methods

### 2.1. Literature Search

This study was conducted according to a predetermined protocol (PROSPERO # CRD42015029597) developed using the Preferred Reporting Items for Systematic Reviews and Meta-Analysis for Protocols [13,14]. We systematically searched PubMed, Embase, and the Cochrane library for eligible randomized, controlled clinical trials published before February 19, 2017 without any language restrictions. The literature search involved querying the terms “coffee,” “tea,” “glucose,” “HbA1c,” “insulin,” “insulin resistance,” and “randomized controlled trial (RCT).” In addition, we manually searched the references included in each original article retrieved. The details of the literature search strategy are described in the Supplementary Table S1.

### 2.2. Study Selection

We included studies examining the effects of caffeinated coffee, decaffeinated coffee, black tea, oolong tea, and green tea (caffeinated or decaffeinated), given as a drink or crude extract, with follow-up durations of at least 2 weeks. We excluded studies examining multi-nutrient supplements, in addition to coffee or tea as interventions. Studies were selected for this analysis only if they also described an RCT in human adults involving either a parallel or crossover design and had at minimum, fasting blood glucose (FBG) or hemoglobin A1c (HbA1c) results available for each group. In cases involving multiple publications of the same study, the most informative article was included.

### 2.3. Data Extraction

Two investigators extracted data independently and resolved any discrepancies through discussion. The extracted information included study characteristics (authors, design, publication year, sample size, duration of intervention, and follow-up), participant characteristics (age, sex, ethnicity, body mass index, HbA1c, FBG concentration), 2-h post-load glucose concentration from an oral glucose tolerance test (75-g OGTT 2h-PG), fasting blood insulin (fasting IRI) concentration, homeostasis model assessment for insulin resistance (HOMA-IR) and HOMA-ß, and intervention (type of tea or coffee).

### 2.4. Risk of Bias Assessment

The risk of bias was assessed using Cochrane’s risk of bias tool [15]. In addition, quality of evidence was assessed using the GRADE system [11].

### 2.5. Strategy for Data Synthesis

We examined the effects of the studied interventions after combining green tea and green tea extract into a single group referred to as green tea. Decaffeinated green tea and decaffeinated green tea extract interventions were also combined into a single decaffeinated green tea group. A standard, pairwise meta-analysis was conducted for each pairwise direct comparison of interventions (caffeinated coffee, decaffeinated coffee, black tea, oolong tea, caffeinated green tea, and decaffeinated green tea). The outcome data (post-intervention values) were extracted for subsequent analyses. When studies reported data for different durations of intervention, the duration for the pre-defined primary endpoint in each study, was used in the analyses. If the standard deviations of the endpoints were missing, pooled standard deviations were calculated from the other included studies [16]. We used a random-effects model to incorporate the assumption that different studies estimated different yet related intervention effects. Publication biases or small study effects were examined using conventional funnel plots.

In addition, we conducted network meta-analyses within the frequentist framework using multivariate random-effects meta-analysis models, which consider the heterogeneity of effects across studies. The results for the comparative effects are presented as mean difference estimates and 95% confidence intervals (CIs). We plotted a comparison-adjusted funnel plot to detect the presence of publication bias in the network meta-analysis. We also evaluated the ranking of the intervention effect, i.e., the most efficacious beverage, second best, third best, etc. Inconsistency between the direct and indirect evidence on the network was evaluated using global and local inconsistency tests [17].

Stata 14 software (Stata, College Station, TX, USA) was used for the analyses. The “metan” package [18] was used to conduct the pairwise random-effects meta-analyses and the “metareg” package [19] was used for the meta-regression analyses; the “network” package [20] was used for the NMAs. The *p*-values < 0.05 were considered statistically significant.

### 2.6. Subgroup or Subset Analysis

In the pairwise direct comparison meta-analyses, the heterogeneity of the intervention effects across studies were investigated using the heterogeneity test and the I^2^ statistic.

The sources of possible clinical heterogeneity were listed a priori, such as age, sex, ethnicity, body mass index, baseline fasting blood glucose level, and the baseline HbA1c level. Among these, data on age, sex, ethnicity, body mass index, baseline fasting blood glucose level were available. These were examined as effect modifiers in the meta-regression or subgroup analyses. Sensitivity analyses were also conducted for assessing possible biases.

## 3. Results

### 3.1. Study Selection and Characteristics

**Figure 1:**
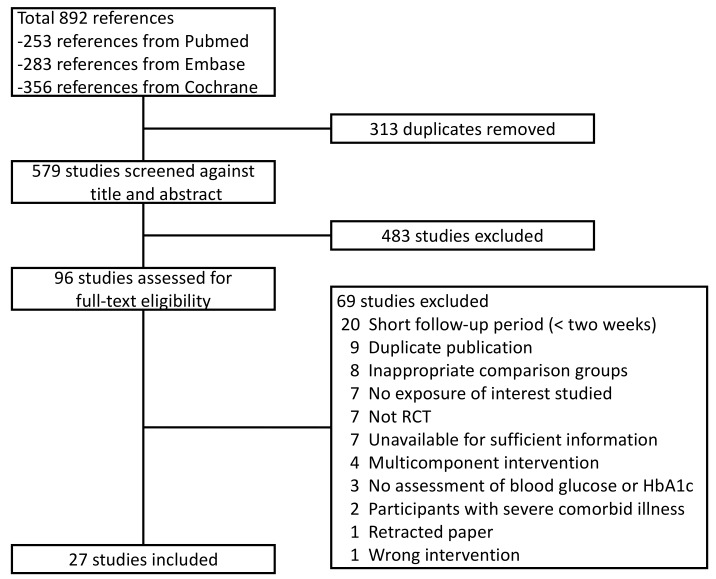
Study selection flow.

**Figure 2:**
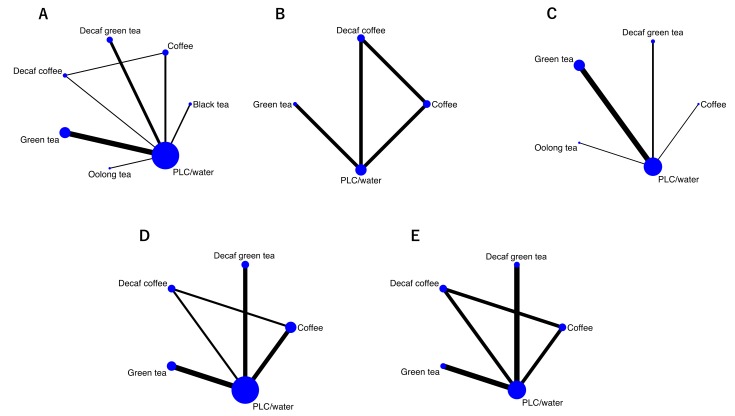
Network maps. (**A**) Fasting blood glucose; (**B**) 2-h post-load glucose concentration from an oral glucose tolerance test; (**C**) HbA1c; (**D**) fasting blood insulin; (**E**) HOMA-IR; Nodes represent the interventions and their sizes represent the number of participants. Edges represent the available direct comparisons between pairs of interventions; the width represents the number of studies comparing the pair of interventions. Abbreviations: Decaf, decaffeinated; PLC, placebo.

Of the 892 citations identified, 27 studies involving 1898 subjects were included in our meta-analysis (Figure 1) [21,22,23,24,25,26,27,28,29,30,31,32,33,34,35,36,37,38,39,40,41,42,43,44,45,46,47]. The characteristics of the included studies are shown in Table 1. Of these studies, effects were reported on FBG concentrations (26 studies), 75-g OGTT 2h-PG concentrations (4 studies), HbA1c (10 studies), fasting IRI concentrations (13 studies), and HOMA-IR (8 studies); no studies reported results on HOMA-ß. Figure 2 shows the network plots of eligible comparisons for FBG, 75-g OGTT 2h-PG, HbA1c, fasting IRI, and HOMA-IR. Six classes of interventions (coffee, decaffeinated coffee, green tea, decaffeinated green tea, black tea, oolong tea) had adequate numbers of studies for NMA. The effects of coffee and decaffeinated coffee were indirectly and directly compared. The differences between direct and indirect evidence in closed loops in the network showed no indication of inconsistency.

### 3.2. Risk of Bias for Included Studies

**Figure 3:**
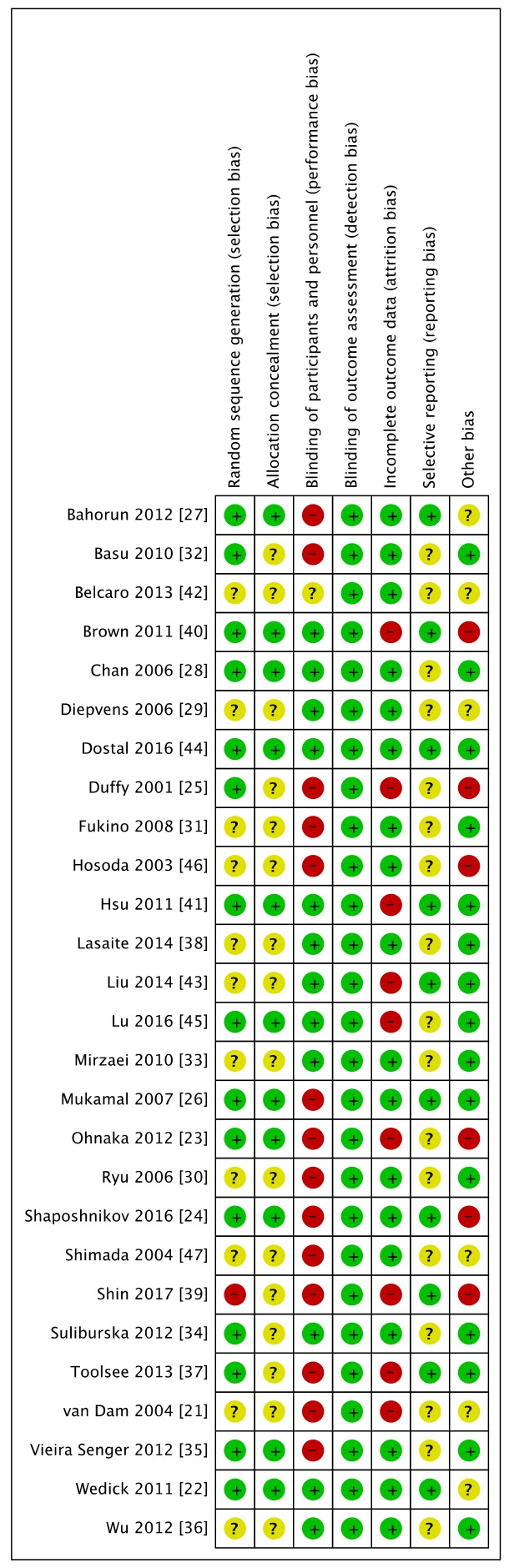
Risk of bias summary.

The included studies generally had unclear or high risks of bias for random sequence generation, allocation concealment, blinding of participants and personnel, blinding of outcome assessments, and selective outcome reporting. The Cochrane risk of bias analysis indicated among the studies with a high risk of bias, blinding of the participants and personnel was highest (51.9%); selective reporting contributed the most to the unclear-domain bias (63.0%). Furthermore, 59.3% of the studies were judged to have an unclear or high risk of bias for either domain or selection bias, which may have seriously distorted the validity of each study. Details regarding the risk of biases are shown in Figure 3.

### 3.3. Primary Endpoint: FBG

Twenty-six studies reported effects on FBG concentrations. Of these, 2 [22,23] reported the effects of both coffee and decaffeinated coffee on FBG concentrations. Thus, the effects on FBG concentrations were reported for coffee (4 studies [21,22,23,24]), decaffeinated coffee (2 studies [22,23]), black tea (3 studies [25,26,27]), green tea (11 studies [28,29,30,31,32,33,34,35,36,37,39]), decaffeinated green tea (6 studies [40,41,42,43,44,45]), oolong tea (2 studies [46,47]).

**Figure 4:**
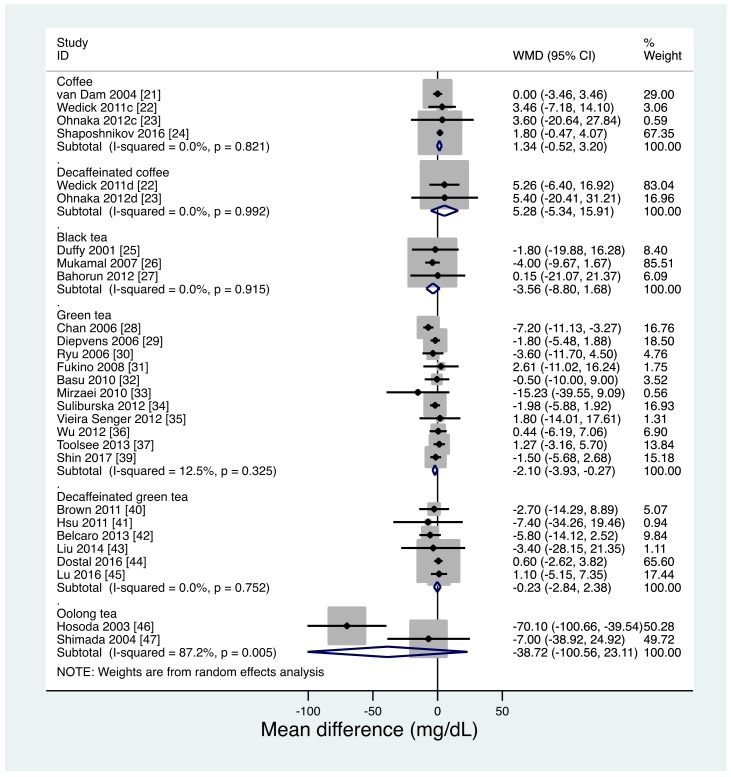
Direct pairwise meta-analysis of effects on fasting blood glucose levels. c, coffee study arm; d, decaffeinated coffee study arm.

A direct pairwise meta-analysis (−2.10 mg/dL; 95% CI, −3.93 to −0.27 mg/dL; *p* = 0.02) and NMA for studies with a moderate quality of evidence (−2.10 mg/dL; 95% CI, −3.96 to −0.24 mg/dL; *p* = 0.03) suggested that green tea consumption slightly but significantly reduced FBG concentrations compared with placebo/water (Table 2, Figure 4).

**Figure 5:**
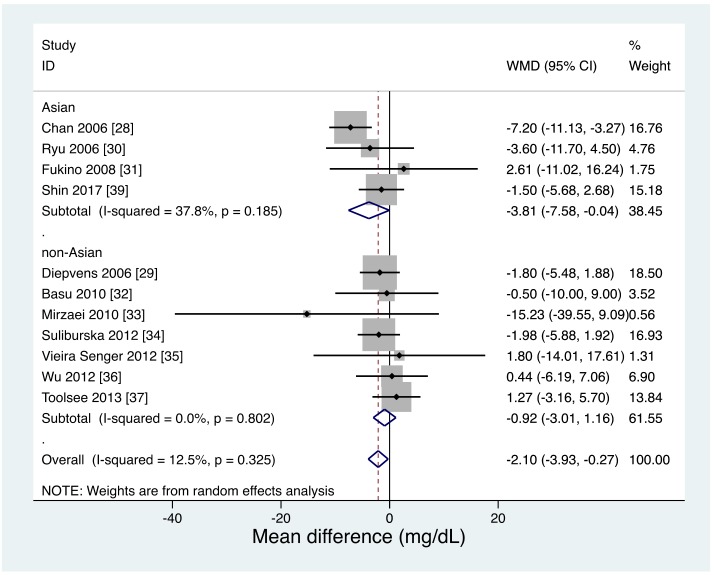
Direct pairwise meta-analysis: effects of green tea on fasting blood glucose levels stratified by Asian-based studies and non-Asian based studies.

The meta-regression analysis showed a tendency for the FBG reductive effect of green tea to be stronger in studies with a mean age < 55-years-old than in studies with an older mean age (β = 0.17; standard error (SE), 0.10; 95% CI, −0.04 to 0.41; *p* = 0.10; Supplemental Figure S1a). In the NMA, stratified by mean participant age, the effect of green tea on FBG concentrations was significant only in younger individuals (age < 55: −2.51 mg/dL; 95% CI, −4.67 to −0.35; *p* = 0.02; age ≥ 55: −0.72 mg/dL; 95% CI, −4.61 to 3.17 mg/dL; *p* = 0.72). In the direct pairwise meta-analysis, stratified by Asian-based studies and non-Asian-based studies, the effect of green tea on FBG concentrations was significant only in Asian-based studies (Asian: −3.81 mg/dL; 95% CI, −7.58 to −0.04; *p* = 0.04; non-Asian: −0.92 mg/dL; 95% CI, −3.01 to 1.16 mg/dL; *p* = 0.39; Figure 5).

Meta-regression analysis indicated that the intervention duration did not modify the effects of green tea on FBG concentrations (β = 0.01; SE, 0.08; 95% CI, −0.16 to 0.19; *p* = 0.87; Supplemental Figure S1b). Meta-regression analysis also indicated that the percentage of male sex, BMI, and baseline FBG did not modify the effects of green tea on FBG concentrations (percentage of male sex: β = 0.04; SE, 0.03; 95% CI, −0.04 to 0.12; *p* = 0.25, BMI: β = −0.20; SE, 0.32; 95%, CI −0.92 to 0.52; *p* = 0.55, baseline FBG: β = −0.01; SE, 0.11; 95% CI, −0.25 to 0.24; *p* = 0.96). In a post-hoc stratified analysis according to non-diabetic [28,29,34,36,39], prediabetic (including subjects with metabolic syndrome) [31,32,35,37], and diabetic subjects [30,33], the significant effect of green tea on FBG concentrations was only evident in a subgroup with non-diabetic subjects (non-diabetic: −2.74 mg/dL; 95% CI, −5.19 to −0.29 mg/dL; *p* = 0.03, prediabetic: 1.13 mg/dL; 95% CI, −2.62 to 4.87 mg/dL; *p* = 0.56, diabetic: −4.76 mg/dL; 95% CI, −12.45 to 2.92 mg/dL; *p* = 0.23; Supplemental Figure S1c). Further, a post-hoc meta-regression analysis suggested that prediabetes or diabetes did not modify the effects of green tea on FBG concentrations (meta-regression heterogeneity; prediabetes: *p* = 0.24, diabetes: *p* = 0.64).

The oolong tea group also had lower FBG concentrations than did the water group in the NMA (−39.91 mg/dL; 95% CI, −62.04 to −17.78 mg/dL; *p* < 0.001; Table 2) but not in the direct pairwise meta-analysis. However, the quality of evidence was very low because of a serious risk of bias due to unclear allocation concealment in all oolong tea studies and a potential financial bias, serious heterogeneity (I^2^ of 87.2%), and very serious imprecision due to having a small sample size and wide confidence interval. Coffee, decaffeinated coffee, black tea, and decaffeinated green tea did not show significant effects on FBG concentrations in either the direct pairwise meta-analysis or the NMA.

Ranking the effects of the interventions on FBG concentrations, after NMA, showed that oolong tea had a mean rank of 1.0 (surface under the cumulative ranking curve (SUCRA), 1.0), black tea had a mean rank of 2.8 (SUCRA, 0.7), and green tea had a mean rank of 3.8 (SUCRA, 0.7). The oolong tea intervention had a one-order larger effect on FBG concentrations as compared with the other interventions, although the quality of evidence was very low. The resulting large heterogeneity in effect sizes across the interventions may have led to unstable NMA estimates. However, excluding the oolong tea studies did not materially change the NMA results (Supplemental Table S2). As shown in the comparison-adjusted funnel plot (Supplemental Figure S2), there was no apparent evidence of asymmetry.

### 3.4. Secondary Endpoints:

#### 3.4.1. 75-g OGTT 2h-PG

Four studies [22,23,28,33] reported the effects of the interventions on 75-g OGTT 2h-PG concentrations, including two studies [28,33] involving green tea and two [22,23] involving both coffee and decaffeinated coffee. Neither the direct pairwise meta-analysis nor the NMA showed any significant effects associated with coffee, decaffeinated coffee, or green tea on 75-g OGTT 2h-PG concentrations relative to a placebo/water (Table 3, Supplemental Figure S3). The comparison-adjusted funnel plot (Supplemental Figure S4) does not show any evidence of asymmetry.

#### 3.4.2. HbA1c

Ten studies reported the interventions’ effects on HbA1c, including 1 [24] showing the effects of coffee, six [31,32,33,36,37,38] showing the effects of green tea, 2 [41,43] showing the effects of decaffeinated green tea, and 1 [47] showing the effects of oolong tea. Thus, we conducted meta-analyses examining green tea (6 studies [31,32,33,36,37,38]) and decaffeinated green tea (2 studies [41,43]) vs. placebo/water. Neither direct pairwise meta-analysis nor NMA showed any significant effect for these interventions on HbA1c (Table 4, Supplemental Figure S5). The comparison-adjusted funnel plot (Supplemental Figure S6) does not show any asymmetry related to green tea.

#### 3.4.3. Fasting IRI

Fifteen studies [21,22,23,24,28,30,31,33,36,40,41,42,43] reported the effects of the interventions on fasting IRI concentrations relative to a placebo/water, including two [21,24] involving coffee, two [22,23] involving both coffee and decaffeinated coffee, five [28,30,31,33,36] involving green tea, and four [40,41,43] involving decaffeinated green tea. Only coffee showed significant impacts on fasting IRI concentrations, in both the direct pairwise meta-analyses (+1.1 μIU/mL; 95% CI, 0.22.0 μIU/mL; Supplemental Figure S7) and in the NMA (+1.1 μIU/mL; 95% CI, 0.2–2.0 μIU/mL; Table 5). However, the comparison-adjusted funnel plot (Supplemental Figure S8) did not show any evidence of asymmetry.

#### 3.4.4. HOMA-IR

Eight studies [22,23,30,31,32,41,42,43] reported the effects of the interventions on HOMA-IR, including two involving both coffee and decaffeinated coffee [22,23], 3 [30,31,32] involving green tea, and three [41,42,43] involving decaffeinated green tea. There were no significant effects of these interventions on HOMA-IR levels relative to placebo/water in the direct pairwise meta-analysis or in the NMA (Table 6, Supplemental Figure S9). Additionally, the comparison-adjusted funnel plot (Supplemental Figure S10) did not show any evidence of asymmetry.

## 4. Discussion

In this systematic review and NMA, we evaluated the effects of various coffee and tea consumptions on glucose metabolism across available RCT data. We found 27 studies, involving 1898 subjects with study durations of 4–72 weeks. With regard to the primary endpoint, the studies with a moderate quality of evidence suggested that green tea consumption, but not consumption of caffeinated/decaffeinated coffee or black tea, may reduce FBG concentrations, as compared with a placebo/water. The effect estimates were also statistically significant for oolong tea, but the quality of evidence was very low due to the risk of bias and imprecision in the studies. As for the secondary endpoint, studies with a moderate quality of evidence indicated that caffeinated coffee consumption may increase insulin concentrations. The potential effects of green tea on glucose metabolism have substantial public health implications given the global diabetes epidemic, such that even a small potential downward shift in the distribution of FBG concentrations would result in substantial numbers of individuals avoiding diabetes. Although further efforts are required to confirm the evidence, our findings support the notion that green tea may be a preventative strategy for reducing the number of people developing diabetes.

Some mechanisms have been suggested to explain the ability of green tea to reduce FBG concentrations. Previous rodent-based studies reported that the potential beneficial effect of green tea on glucose metabolism may be mediated by epigallocatechin gallate (EGCG), the most abundant catechin present in green tea [48]. Waltner-Law et al. reported that EGCG reduces hepatic glucose production by increasing tyrosine phosphorylation of the insulin receptor and insulin receptor substrate-1 in H4IIE rat hepatoma cell models [49]. Recent studies have also suggested that green tea increases insulin sensitivity and glucose metabolism, helping to prevent type 2 diabetes from developing. Ortsäter et al. also reported that EGCG preserves islet structure and enhances glucose tolerance in genetically diabetic mice (young *db*/*db* mice) [50]. Further, Ueda et al. reported that EGCG may reduce hyperglycemic events by promoting glucose transporter-4 translocation in skeletal muscle via a mechanism that is partially different from the action of insulin because EGCG promoted the translocation in insulin-resistant L6 myotubes and had neither a synergistic nor an additive effect on insulin [51]. Surprisingly, Fu et al. reported that EGCG increases the concentrations of the circulating anti-inflammatory cytokine, interleukin-10, and delayed type 1 diabetes onset in non-obese diabetic mice [52]. In our meta-analysis, green tea and its extract reduced FBG concentrations. In the NMA, the effect of green tea on FBG concentrations was confined to younger (< 55-years-old) subjects. Beta-cell function is known to decrease continuously from euglycemia until the onset of type 2 diabetes [53,54]. Therefore, early green tea intervention, which may help maintain beta-cell function, might be a prerequisite for its potential effects on FBG concentrations, at least via the proposed beta-cell protection mechanism. The reason for the discrepancy between results derived from Asian-based studies and non-Asian-based studies is unclear. In both Asian and non-Asian studies, 75% of studies used green tea extract for the intervention. If the EGCG content was not provided, we assumed that one cup of green tea contains 110 mg of EGCG [32] and 100 mg of green tea extract contains 21.4 mg of EGCG [29] from similar studies. The mean daily EGCG doses were not different between the Asian and non-Asian studies (367.3 ± 175.5 mg/day vs. 374.6 ± 186.8 mg/day, *p* = 0.95). The meta-regression analysis showed there was no significant interaction between daily the EGCG dose contained in green tea and the FBG (β = 0.00; SE, 0.01; 95% CI, −0.02 to 0.01; *p* = 0.54; Supplemental Figure S11).

One possible explanation could be the differences in dietary habits or genetic predisposition for impaired glucose metabolism that might interact with the glucose-lowering effects of green tea. However, further investigations are certainly needed to understand this possible ethnic difference. Caffeine is known to reduce insulin sensitivity in the short term and have adverse effects such as arrhythmias, pregnancy complications, and drug interactions from clinical trials [55]. Thus, such potential risks associated with caffeine should also be evaluated in order to make recommendations regarding caffeine-related beverages such as caffeinated coffee and tea. In our meta-analysis, green tea reduced FBG levels but did not reduce HbA1c levels. Two-thirds of the included green tea studies with HbA1c data were followed up in less than 12 weeks. This might be too short a period for assessing the effects for change in HbA1c levels accurately.

Oolong tea also showed potential protective effects on FBG concentrations in the NMA. Both green tea and oolong tea are derived from *Camellia sinensis*, with their only difference being in the level of fermentation; green tea is unfermented whereas oolong tea is partially fermented. Therefore, both may exert protective effects on glucose metabolism through similar mechanisms. However, the two oolong tea studies included in our meta-analysis were supported by oolong tea manufacturers and had a very low quality of evidence due to the presence of very serious risks of bias and serious imprecisions. Thus, our inferences regarding the potential protective effects of oolong tea are limited. Indeed, a five-day, cross-over trial of 19 participants failed to show the protective effects of oolong tea on glucose metabolism, i.e., FBG concentrations and incremental glucose areas under the concentration time curve remained largely unchanged in the trial arms [56,57,58]. To assess the true effects of oolong tea on glucose metabolism, more precisely designed, larger-scale RCTs are required. A series of prospective cohort studies reported that coffee and decaffeinated coffee intakes were linked to reducing the risk of type 2 diabetes [57]. In a meta-analysis and systematic review that summarized the findings from 28 prospective studies, the relative risk of diabetes associated with a 1-cup/day consumption increase was 0.91 (95% CI, 0.89–0.94) for caffeinated coffee consumption and 0.94 (95% CI, 0.91–0.98) for decaffeinated coffee consumption [21]. Further, these findings also suggested a dose-response relationship. However, in our meta-analysis, coffee and decaffeinated coffee consumption were unrelated to FBG concentrations. Furthermore, our meta-analysis showed that coffee resulted in slight increases in fasting IRI concentrations, raising concerns that coffee may impair insulin sensitivity. Because the follow-up periods of the included studies were far shorter than for the cohort studies and the total number of participants of coffee trials was small (below optimal information criterion), the long-term effect of coffee on glucose metabolism remains uncertain and longer (a few years) RCTs with a sufficient number of participants are required.

To date, this meta-analysis is the most comprehensive analysis, using a combined NMA and GRADE approach to evaluate the effects of tea and coffee on glucose metabolism. However, several limitations merit further consideration. First, complete blinding of coffee or tea interventions is difficult due to their taste. Thus, most studies were conducted using an open-label design. Second, the study durations were relatively short (median, 9 weeks; interquartile range, 6.5–16 weeks). Therefore, additional randomized studies, having longer durations and sufficient washout periods, are needed to determine the long-term effects of coffee and tea consumption on glucose metabolism.

## 5. Conclusions

In conclusion, this systematic review and NMA of 27 studies, involving 1898 subjects and having a moderate quality of evidence, indicates that green tea consumption, compared with a placebo/water, might slightly lower FBG concentrations. Oolong tea consumption also resulted in significant decreases in FBG concentrations in the NMA but the studies suffered from a very low quality of evidence. Further, studies with a moderate quality of evidence indicated that caffeinated coffee consumption may increase insulin concentrations. The effects of consuming coffee or tea on HbA1c, 75-g OGTT 2h-PG, HOMA-IR, and HOMA-ß are limited and uncertain. Although further efforts are required to confirm the benefit of green tea on glucose metabolism, our study provides supportive evidence that green tea might lower FBG concentrations, possibly leading to a reduction in the number of people developing diabetes as a result of regular consumption.

## Figures and Tables

**Table 1:** Characteristics of the included studies.

Study ID	Study Design	Population	Country	Interventions	Outcomes	Total Sample Size	Duration of Intervention (weeks)	Age	Male Sex (%)	BMI (kg/m^2^)	Baseline FBG (mg/dL)
van Dam 2004 [21]	Crossover	Healthy volunteers	Netherlands	Coffee/no coffee	FBG, F-IRI	26	4	37.0	38	23.0	NR
Wedick 2011 [22]	Parallel	Overweight	US	Coffee/decaf coffee/no coffee	FBG, 2hPG, F-IRI, IR	45	8	40.6	35	29.5	86.4
Ohnaka 2012 [23]	Parallel	Overweight men with IFG	Japan	Coffee/decaf coffee/water	FBG, 2hPG, F-IRI, IR	43	16	52.7	100	25-30	107.6
Shaposhnikov 2016 [24]	Parallel	Healthy volunteers	Germany	Coffee/water	FBG, A1c, F-IRI,	160	8	51.0	NR	26.5	97.2
Duffy 2001 [25]	Crossover	CAD	US	Black tea/water	FBG	50	4	55.0	78	29.7	121.5
Mukamal 2007 [26]	Parallel	Diabetes or 2 other cardiovascular risk factors	US	Black tea/water	FBG	28	26	65.8	36	29.1	97.0
Bahorun 2012 [27]	Parallel	Healthy subjects	Mauritius	Black tea/water	FBG	77	12	25–74	55	NR	125.5
Chan 2006 [28]	Parallel	Obese women with polycystic ovary syndrome	China	Green tea extract/placebo	FBG, 2hPG, F-IRI	34	13	25–40	0	30.1	92.6
Diepvens 2006 [29]	Parallel	Overweight female	Netherlands	Green tea extract/placebo	FBG	46	12	41.7	0	27.7	93.6
Ryu 2006 [30]	Crossover	T2D	Korea	Green tea/water	FBG, F-IRI, IR	55	4	53.9	56	25.0	NR
Fukino 2008 [31]	Crossover	Prediabetes	Japan	Green tea extract/water	FBG, A1c, F-IRI, IR	60	9	53.6	85	25.7	137.7
Basu 2010 [32]	Parallel	Obesity & MetS	US	Green tea/green tea extract/water	FBG, A1c, IR	35	8	42.5	49	36.2	88.2
Mirzaei 2010 [33]	Parallel	T2D	Iran	Green tea extract/placebo	FBG, 2hPG, A1c, F-IRI	102	8	54.6	21	29.2	172.1
Suliburska 2012 [34]	Crossover	Obese	Poland	Green tea extract/placebo	FBG	46	13	50.4	50	32.8	101.8
Vieira Senger 2012 [35]	Parallel	MetS	Brazil	Green tea/no green tea	FBG	45	9	≥60	16	30.5	119.0
Wu 2012 [36]	Parallel	Postmenopausal women	US	Green tea extract/placebo	FBG, A1c, F-IRI,	103	9	59.8	0	29.3	99.7
Toolsee 2013 [37]	Parallel	Prediabetes	Mauritius	Green tea/water	FBG, A1c,	117	14	48.3	51	25.6	91.6
Lasaite 2014 [38]	Parallel	T2D	Lithuania	Green tea extract/placebo	A1c	31	78	57.0	38	NR	NR
Shin 2017 [39]	Parallel	After endoscopic polypectomy	Korea	Green tea extract/no Green tea extract	FBG	143	52	59.7	68	23.9	101.7
Brown 2011 [40]	Crossover	Healthy overweight and obese men	UK	Decaf green tea extract/placebo	FBG, F-IRI	66	6	49.4	100	31.5	107.1
Hsu 2011 [41]	Parallel	T2D	Taiwan	Decaf green tea extract/placebo	FBG, A1c, F-IRI, IR	68	16	51.3	35	29.8	173.0
Belcaro 2013 [42]	Parallel	MetS	Italia	Decaf green tea extract/placebo	FBG	98	24	46.5	50	31.0	115.9
Liu 2014 [43]	Parallel	T2D with dyslipidemia	Taiwan	Decaf green tea extract/placebo	FBG, A1c, F-IRI, IR	77	16	54.3	42	26.3	145.6
Dostal 2016 [44]	Parallel	Obese women	US	Decaf green tea extract/placebo	FBG, F-IRI, IR	237	52	60.7	0	28.2	97.4
Lu 2016 [45]	Parallel	Women with acne	Taiwan	Decaf green tea extract/placebo	FBG	64	4	29.1	0	21.2	87.6
Hosoda 2003 [46]	Crossover	T2D	Taiwan	Oolong tea/water	FBG	20	4	61.2	50	22.6	NR
Shimada 2004 [47]	Crossover	CAD	Japan	Oolong tea/water	FBG, A1c	22	4	64.3	77	23.0	170.5

Abbreviations: 2hPG; 75-g 2-h oral glucose tolerance test results for blood glucose; A1c, HbA1c; BMI, body mass index; CAD, coronary artery disease; F-IRI, fasting blood insulin; FBG, fasting blood glucose; IFG, impaired fasting glucose; IR, HOMA-IR; MetS: metabolic syndrome; NR, not reported; T2D, type 2 diabetes.

**Table 2:** Intervention effect on fasting blood glucose vs. placebo/water.

Intervention	Number of Studies in Pairwise Comparison	Number of Participants in Pairwise Comparison	Mean Difference (95% CI, mg/dL)	I^2^ (%)	Quality of Evidence
Coffee (pairwise)	4	247	1.34 (−0.52 to 3.20)	0.0	Low ^a^
Coffee (NMA)			1.27 (−1.18 to 3.71)		
Decaffeinated coffee (pairwise)	2	55	5.28 (−5.34 to 15.91)	0.0	Low ^b^
Decaffeinated coffee (NMA)			4.12 (−5.41 to 13.65)		
Black tea (pairwise)	3	155	−3.56 (−8.80 to 1.68)	0.0	Low ^c^
Black tea (NMA)			−3.51 (−9.09 to 2.07)		
Green tea (pairwise)	11	786	−2.10 (−3.93 to −0.27)	12.5	Moderate ^d^
Green tea (NMA)			−2.09 (−3.96 to −0.24)		
Decaffeinated green tea (pairwise)	6	610	−0.23 (−2.84 to 2.38)	0.0	Low ^e^
Decaffeinated green tea (NMA)			−0.44 (−3.53 to 2.64)		
Oolong tea (pairwise)	2	42	−38.72 (−100.56 to 23.11)	87.2	Very low ^f^
Oolong tea (NMA)			−39.91 (−62.04 to −17.78)		

^a^ Downgraded two levels because of a serious risk of bias due to unclear allocation concealment in studies with large weight and serious imprecision due to having a small sample size (below optimal information criterion). ^b^ Downgraded two levels because of very serious imprecision due to having a small sample size (below optimal information criterion) and wide confidence interval. ^c^ Downgraded two levels because of serious risk of bias in blinding of participants in all studies and serious imprecision due to having a small sample size (below optimal information criterion). ^d^ Downgraded one level because of serious risk of bias due to unclear allocation concealment in studies with large weight. ^e^ Downgraded two levels because of serious risk of bias due to unclear allocation concealment in studies with large weight and serious imprecision due to having a small sample size (below optimal information criterion). ^f^ Downgraded three levels because of serious risk of bias due to unclear allocation concealment in all studies and potential financial bias; serious heterogeneity (I^2^ of 87.2%) and very serious imprecision due to having a small sample size (below optimal information criterion) and wide confidence intervals.

**Table 3:** Intervention effect on 2-h oral glucose tolerance test results for blood glucose vs. placebo/water.

Intervention	Number of Studies	Number of Intervention	Mean Difference (95% CI) (mg/dL)	I^2^ (%)	Quality of Evidence
Coffee (pairwise)	2	61	−23.99 (−63.78 to 15.81)	66.4	Very low ^a^
Coffee (NMA)			−17.89 (−44.95 to 9.18)		
Decaffeinated coffee (pairwise)	2	55	12.27 (−8.52 to 33.07)	0.0	Low ^b^
Decaffeinated coffee (NMA)			12.20 (−11.33 to 35.73)		
Green tea (pairwise)	2	138	−8.25 (−27.11 to 10.61)	0.0	Moderate ^c^
Green tea (NMA)			−8.35 (−29.40 to 12.70)		

^a^ Downgraded three levels because of serious risk of bias due to inadequate allocation concealment in all studies (designed as coffee vs no coffee), high heterogeneity (I^2^ = 66.4), serious imprecision due to having a small sample size, and wide confidence interval. ^b^ Downgraded two levels because of serious risk of bias due to distinguishable allocation in all studies (designed as decaffeinated coffee vs no coffee), serious imprecision due to having a small sample size, and wide confidence interval. ^c^ Downgraded one level because of a small sample size (below optimal information criterion) and wide confidence interval.

**Table 4:** Intervention effect on HbA1c vs. placebo/water.

Intervention	Number of Studies	Number of Interventions	Mean Difference (95% CI) (mg/dL)	I^2^ (%)	Quality of Evidence
Green tea (pairwise)	6	504	0.00 (−0.15 to 0.16)	23.8	Low ^a^
Green tea (NMA)			0.02 (−0.09 to 0.12)		
Decaffeinated green tea (pairwise)	2	145	−0.08 (−0.67 to 0.51)	0.0	Moderate ^b^
Decaffeinated green tea (NMA)			−0.08 (−0.67 to 0.51)		

^a^ Downgraded two levels because of serious risk of bias due to unclear allocation concealment and serious imprecision due to having a small sample size (below optimal information criterion). ^b^ Downgraded one level because of serious imprecision due to having a small sample size (below optimal information criterion).

**Table 5:** Intervention effect on fasting blood insulin vs. placebo/water.

Intervention	Number of Studies	Number of Interventions	Mean Difference (95% CI) (mg/dL)	I^2^ (%)	Quality of Evidence
Coffee (pairwise)	4	273	1.10 (0.17 to 2.03)	0.0	Low ^a^
Coffee (NMA)			1.10 (0.17 to 2.04)		
Decaffeinated coffee (pairwise)	2	55	0.00 (−4.99 to 5.00)	46.9	Very low ^b^
Decaffeinated coffee (NMA)			1.24 (−1.70 to 4.18)		
Green tea (pairwise)	5	469	−0.11 (−0.67 to 0.45)	0.0	Low ^c^
Green tea (NMA)			−0.11 (−0.67 to 0.45)		
Decaffeinated green tea (pairwise)	4	510	−0.51 (−2.16 to 1.13)	45.7	Moderate ^d^
Decaffeinated green tea (NMA)			−0.02 (−0.83 to 0.80)		

^a^ Downgraded two levels because of serious risk of bias due to blinding of participants and personnel and serious imprecision due to having a small sample size (below optimal information criterion). ^b^ Downgraded three levels because of serious risk of bias due to blinding of participants and personnel, moderate heterogeneity (I^2^ = 46.9), serious imprecision due to having a small sample size (below optimal information criterion), and wide confidence interval. ^c^ Downgraded two levels because of serious risk of bias due to unclear allocation concealment in 4 out of 5 studies and serious imprecision due to having a small sample size (below optimal information criterion). ^d^ Downgraded one level because of moderate heterogeneity (I^2^ = 45.7) and small sample size (below optimal information criterion).

**Table 6:** Intervention effect on HOMA-IR vs. placebo/water.

Intervention	Number of Studies	Number of Interventions	Mean Difference (95% CI)	I^2^ (%)	Quality of Evidence
Coffee (pairwise)	2	61	0.04 (−0.75 to 0.83)	0.0	Low ^a^
Coffee (NMA)			0.05 (−0.79 to 0.89)		
Decaffeinated coffee (pairwise)	2	55	0.14 (−1.13 to 1.41)	35.0	Very low ^b^
Decaffeinated coffee (NMA)			0.18 (−0.83 to 1.20)		
Green tea (pairwise)	3	265	−0.11 (−0.62 to 0.39)	0.0	Low ^c^
Green tea (NMA)			−0.10 (−0.68 to 0.48)		
Decaffeinated green tea (pairwise)	3	382	−0.08 (−1.12 to 0.95)	63.3	Very low ^d^
Decaffeinated green tea (NMA)			0.01 (−0.63 to 0.66)		

^a^ Downgraded two levels because of serious risk of bias due to blinding of participants and serious imprecision due to small sample size (below optimal information criterion). ^b^ Downgraded three levels because of serious risk of bias due to blinding of participants, inconsistency of results, and serious imprecision due to small sample size (below optimal information criterion). ^c^ Downgraded two levels because of serious risk of bias due to blinding of participants and serious imprecision due to small sample size (below optimal information criterion). ^d^ Downgraded three levels because of serious risk of bias due to unclear allocation concealment, inconsistency of results, serious imprecision due to small sample size (below optimal information criterion), and wide confidence interval. HOMA-IR, homeostasis model assessment for insulin resistance.

## Supplementary Materials

The following are available online at http://www.mdpi.com/2072-6643/11/1/48/s1, Search strategy, supplemental Figure S1a: Meta-regression graph between effects of green tea on fasting blood glucose and study mean age; Figure S1b: Meta-regression graph between effects of green tea on fasting blood glucose and intervention duration (weeks); Figure S1c: Direct pairwise meta-analysis, effects of green tea on fasting blood glucose levels, stratified by non-diabetic, prediabetic, and diabetic subjects; Figure S2: Comparison-adjusted funnel plot of effects on fasting blood glucose level; Figure S3: Direct pairwise meta-analysis forest plot of effects on 2-h oral glucose tolerance test results for blood glucose vs. placebo/water; Figure S4: Comparison-Adjusted Funnel Plot of effects on 2-h oral glucose tolerance test results for blood glucose vs. placebo/water; Figure S5: Direct pairwise meta-analysis forest plot of effects on HbA1c vs. placebo/water; Figure S6: Comparison-Adjusted Funnel Plot of effects on HbA1c vs. placebo/water; Figure S7: Direct pairwise meta-analysis forest plot of effects on fasting blood insulin vs. placebo/water; Figure S8: Comparison-Adjusted Funnel Plot of effects on fasting blood insulin vs. placebo/water; Figure S9: Direct pairwise meta-analysis forest plot of effects on HOMA-IR vs. placebo/water; Figure S10: Comparison-Adjusted Funnel Plot of effects on HOMA-IR vs. placebo/water; Figure S11: Meta-regression graph between effects of green tea on fasting blood glucose and daily EGCG dose (mg); Table S1 Search strategy, Table S2 Effects on fasting blood glucose vs. placebo/water, analyzed without oolong tea.
