# Supplementary material for: Effects of Coffee and Tea Consumption on Glucose Metabolism: A Systematic Review and Network Meta-Analysis
Source: Nutrients. 2018 Dec 27;11(1):48. doi: 10.3390/nu11010048 (PMC6356434; doi:10.3390/nu11010048)
Supplement: Supplementary file 1 [file nutrients-11-00048-s001.pdf]

Supplementary Materials

Supplemental Tables

Supplemental Table 1. Search strategy

Supplemental Table 2. Effects on fasting blood glucose vs. placebo/water, analyzed without oolong tea

Supplemental Figures

Supplemental Figure 1a. Meta-regression graph between effects of green tea on fasting blood glucose and study mean age

Supplemental Figure 1b. Meta-regression graph between effects of green tea on fasting blood glucose and intervention duration (weeks)

Supplemental Figure 1c. Direct pairwise meta-analysis, effects of green tea on fasting blood glucose levels, stratified by non-diabetic, prediabetic, and diabetic subjects.

Supplemental Figure 2. Comparison-adjusted funnel plot of effects on fasting blood glucose level.

Supplemental Figure 3. Direct pairwise meta-analysis forest plot of effects on 2-hour oral glucose tolerance test results for blood glucose vs. placebo/water

Supplemental Figure 4. Comparison-Adjusted Funnel Plot of effects on 2-hour oral glucose tolerance test results for blood glucose vs. placebo/water

Supplemental Figure 5. Direct pairwise meta-analysis forest plot of effects on HbA1c vs. placebo/water

Supplemental Figure 6. Comparison-Adjusted Funnel Plot of effects on HbA1c vs. placebo/water

Supplemental Figure 7. Direct pairwise meta-analysis forest plot of effects on fasting blood insulin vs. placebo/water

Supplemental Figure 8. Comparison-Adjusted Funnel Plot of effects on fasting blood insulin vs. placebo/water

Supplemental Figure 9. Direct pairwise meta-analysis forest plot of effects on HOMA-IR vs. placebo/water

Supplemental Figure 10. Comparison-Adjusted Funnel Plot of effects on HOMA-IR vs. placebo/water

Supplemental Figure 11. Meta-regression graph between effects of green tea on fasting blood glucose and daily EGCG dose (mg)

Supplementary Materials

Supplemental Table 1. Search strategy

Search engine	Search strategy
EMBASE	'tea'/exp OR 'green tea extract'/exp OR 'camellia sinensis'/exp OR 'coffee'/exp OR tea OR 'green tea' OR 'black tea' OR 'camellia sinensis' OR coffee AND ('glucose blood level'/exp OR 'insulin blood level'/exp OR (glucose* OR insulin*) NEAR/3 (level* OR blood*) OR 'hemoglobin a1c'/exp OR (hemoglobin OR haemoglobin) NEXT/2 a1c OR 'glycosylated hemoglobin'/exp OR (glycosylated OR glycated OR glycaeted) NEAR/2 (hemoglobin* OR haemoglobin*) OR 'insulin sensitivity'/exp OR 'insulin resistance'/exp OR insulin* NEAR/2 (sensitivit* OR resistance*)) AND ('randomized controlled trial'/exp OR 'randomized controlled trial (topic)'/exp OR random*:ab,ti OR rct:ab,ti) AND [embase]/lim
PUBMED	(("coffee"[MeSH Terms] OR "coffee"[All Fields] OR "tea"[MeSH Terms] OR "tea"[All Fields] OR "camellia sinensis"[MeSH Terms] OR ("camellia"[All Fields] AND "sinensis"[All Fields]) OR "camellia sinensis"[All Fields]) AND ("blood glucose"[MeSH Terms] OR "glucose"[All Fields] OR "hemoglobin a, glycosylated"[MeSH Terms] OR "glycosylated hemoglobin a"[All Fields] OR "glycosylated haemoglobin a"[All Fields] OR "hba1c"[All Fields] OR ("hemoglobin"[All Fields] AND "a1c"[All Fields]) OR ("haemoglobin"[All Fields] AND "a1c"[All Fields]) OR ("glycosylated"[All Fields] AND "hemoglobin"[All Fields]) OR ("glycosylated"[All Fields] AND "haemoglobin"[All Fields]) OR ("glycated"[All Fields] AND "hemoglobin"[All Fields]) OR ("glycated"[All Fields] AND "haemoglobin"[All Fields]) OR ("glycaeted"[All Fields] AND "hemoglobin"[All Fields]) OR ("glycaeted"[All Fields] AND "haemoglobin"[All Fields]) OR "insulin/blood"[Mesh Terms] OR "insulin resistance"[MeSH Terms] OR "insulin resistance"[All Fields] OR ("insulin"[All Fields] AND "resistance"[All Fields]) OR "insulin sensitivity"[All Fields] OR ("insulin"[All Fields] AND "sensitivity"[All Fields])) AND ("randomized controlled trial"[Publication Type] OR "controlled clinical trial"[Publication Type] OR "controlled clinical trial"[All Fields] OR randomized[Title/Abstract] OR randomised[Title/Abstract] OR

	placebo[Title/Abstract] OR "clinical trials as topic"[MeSH Terms] OR "cross-over studies"[MeSH Terms] OR "cross-over studies"[All Fields] OR "cross over studies"[All Fields] OR "Cross-over study"[All Fields] OR "Cross over study"[All Fields] OR "clinical trial"[Publication Type]))

Supplemental Table 2. Effects on fasting blood glucose vs. placebo/water, analyzed without oolong tea

Intervention	Mean difference (95 % CI, mg/dL)	Quality of evidence
Coffee (NMA)	1.27 (–1.14 to 3.68)	Low
Decaffeinated coffee (NMA)	4.12 (–5.40 to 13.64)	Low
Black tea (NMA)	–3.51 (–9.07 to 2.04)	Low
Green tea (NMA)	–2.10 (–3.95 to –0.26)	Moderate
Decaffeinated green tea (NMA)	–0.43 (–3.49 to 2.63)	Low

NMA, network meta-analysis

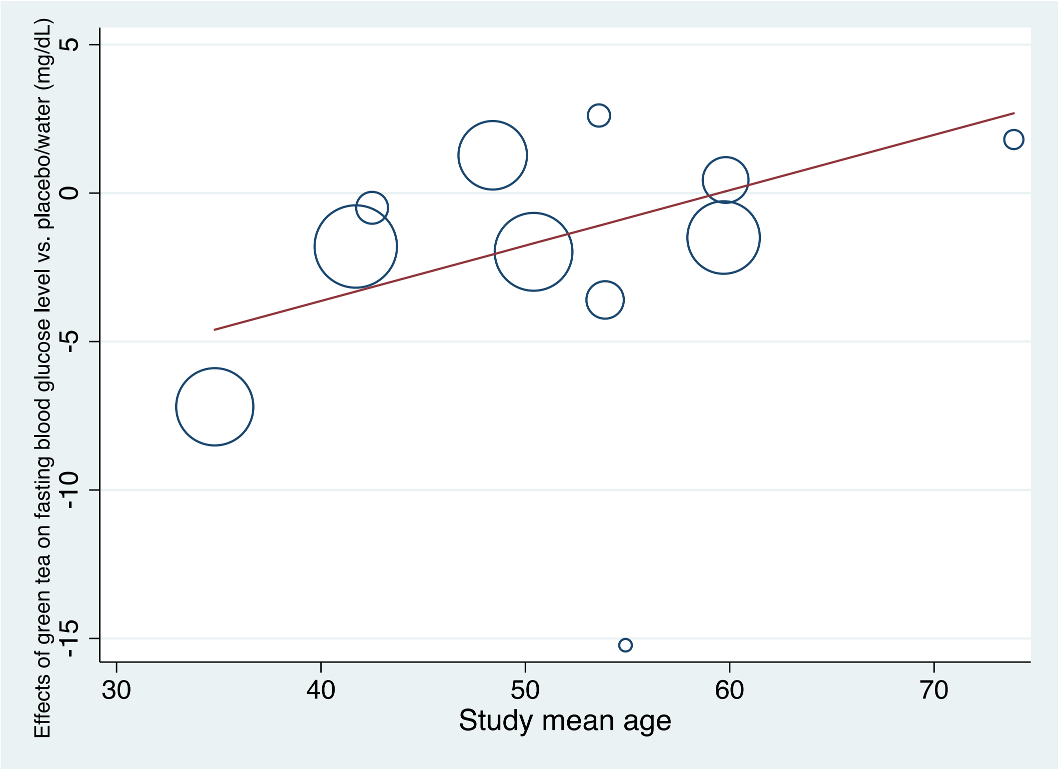

Supplemental Figure 1a. Meta-regression graph between effects of green tea on fasting blood glucose and study mean age (β = 0.17, SE 0.10, 95% CI –0.04 to 0.41, P = 0.10)

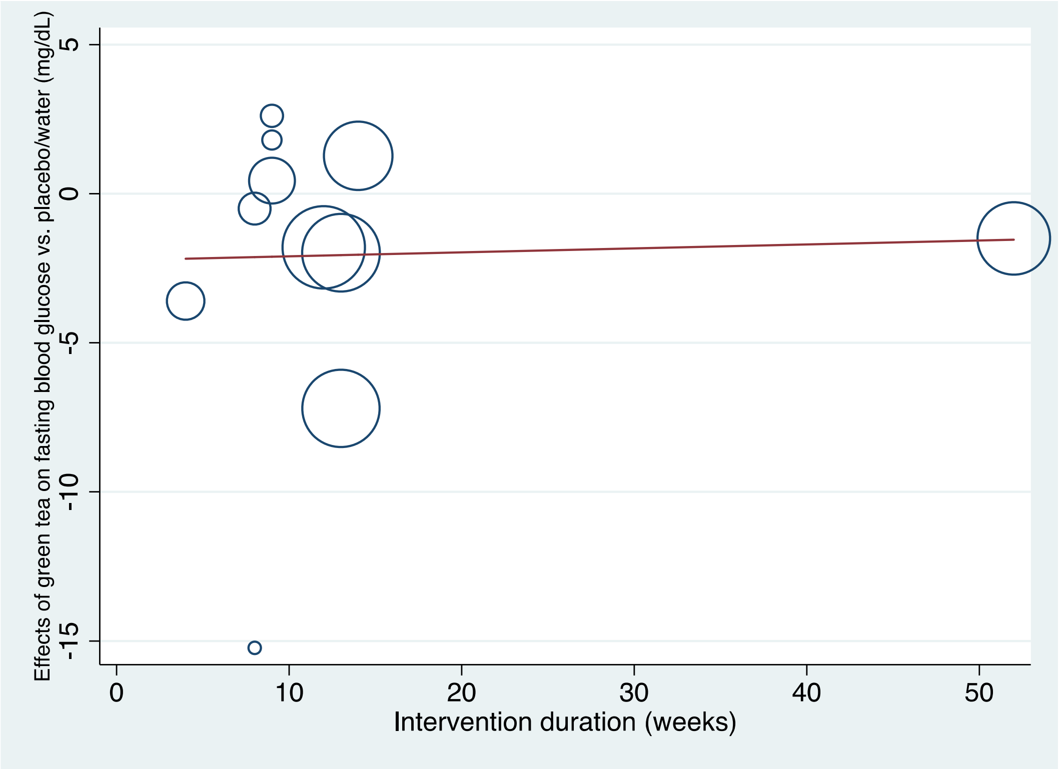

Supplemental Figure 1b. Meta-regression graph between effects of green tea on fasting blood glucose and intervention duration (weeks) (β = 0.01, SE 0.08, 95% CI –0.16 to 0.19, P = 0.87)

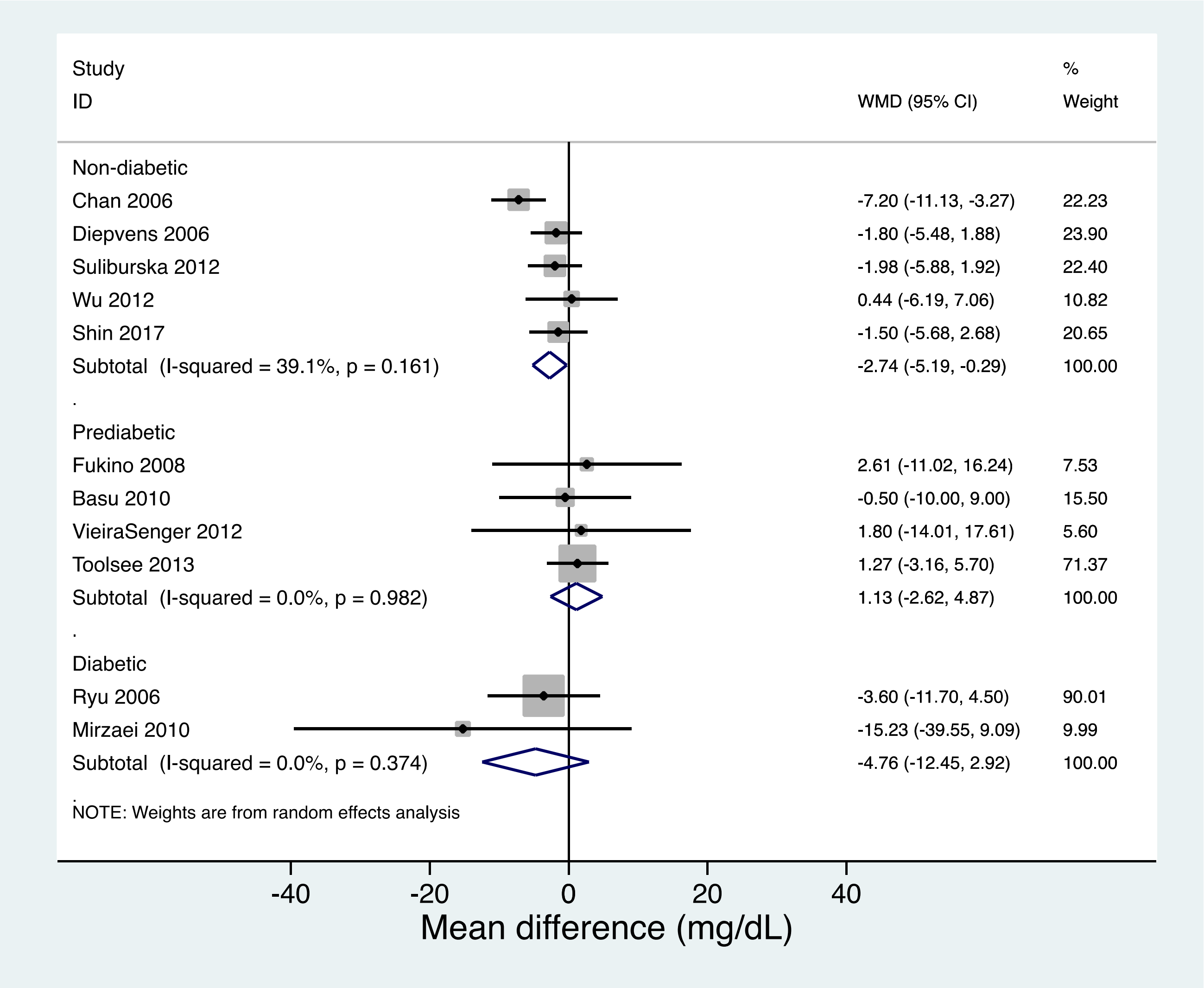

Supplemental Figure 1c. Direct pairwise meta-analysis, effects of green tea on fasting blood glucose levels, stratified by non-diabetic, prediabetic, and diabetic subjects. WMD, weighted mean difference

Supplementary Materials

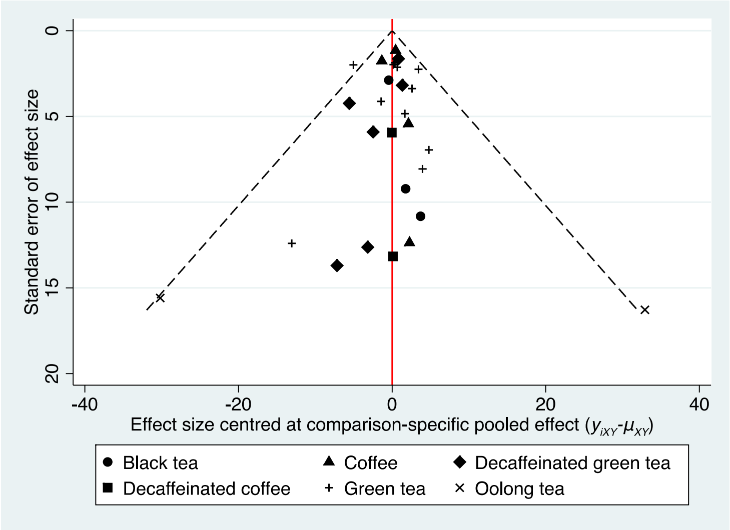

Supplemental Figure 2. Comparison-adjusted funnel plot of effects on fasting blood glucose level.

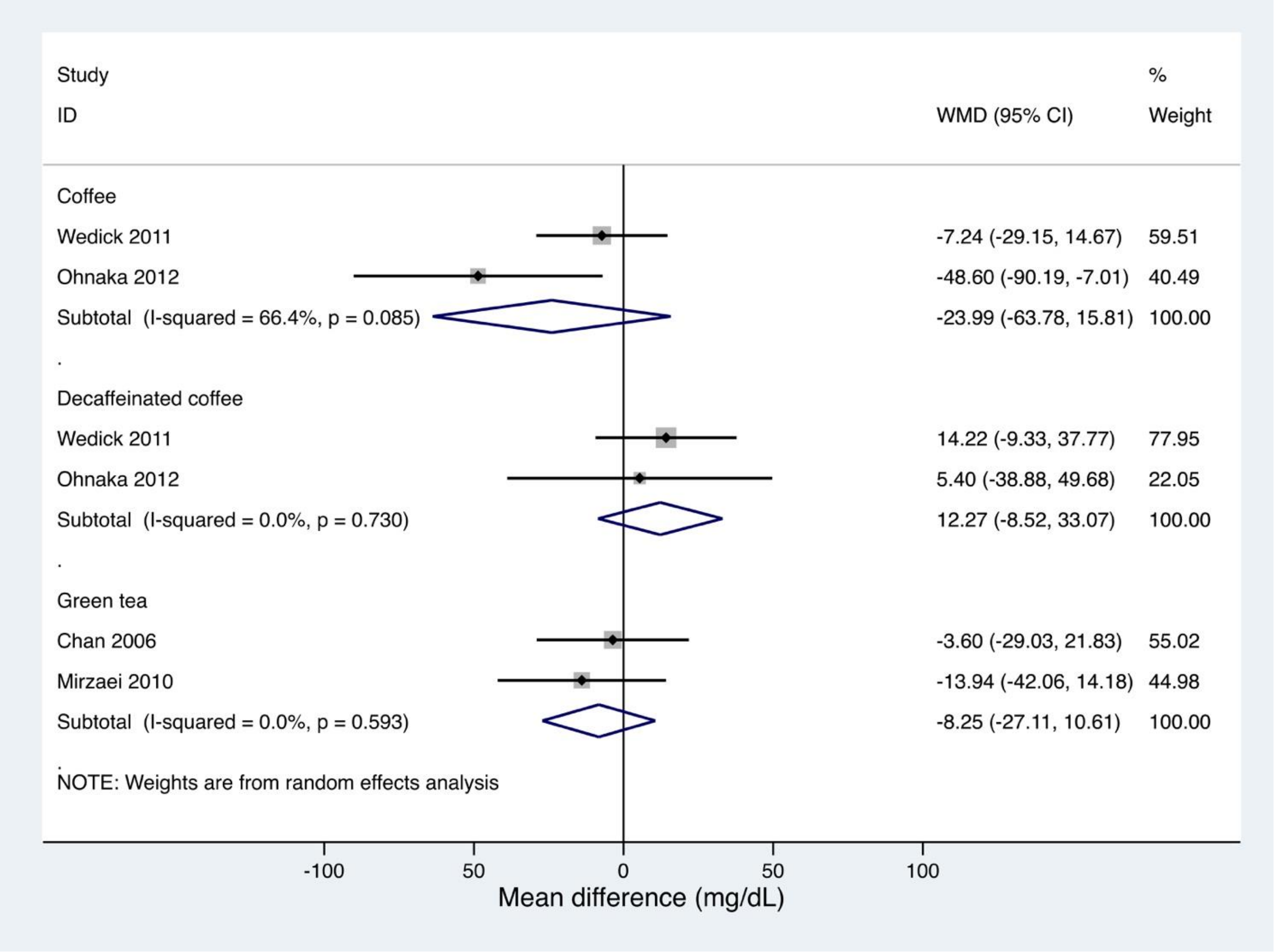

Supplemental Figure 3. Direct pairwise meta-analysis forest plot of effects on 2-hour oral glucose tolerance test results for blood glucose vs. placebo/water. WMD, weighted mean difference

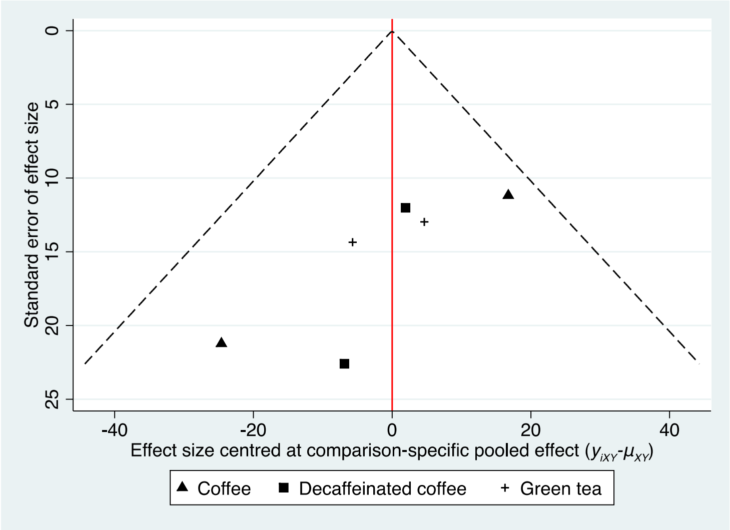

Supplemental Figure 4. Comparison-Adjusted Funnel Plot of effects on 2-hour oral glucose tolerance test results for blood glucose vs. placebo/water.

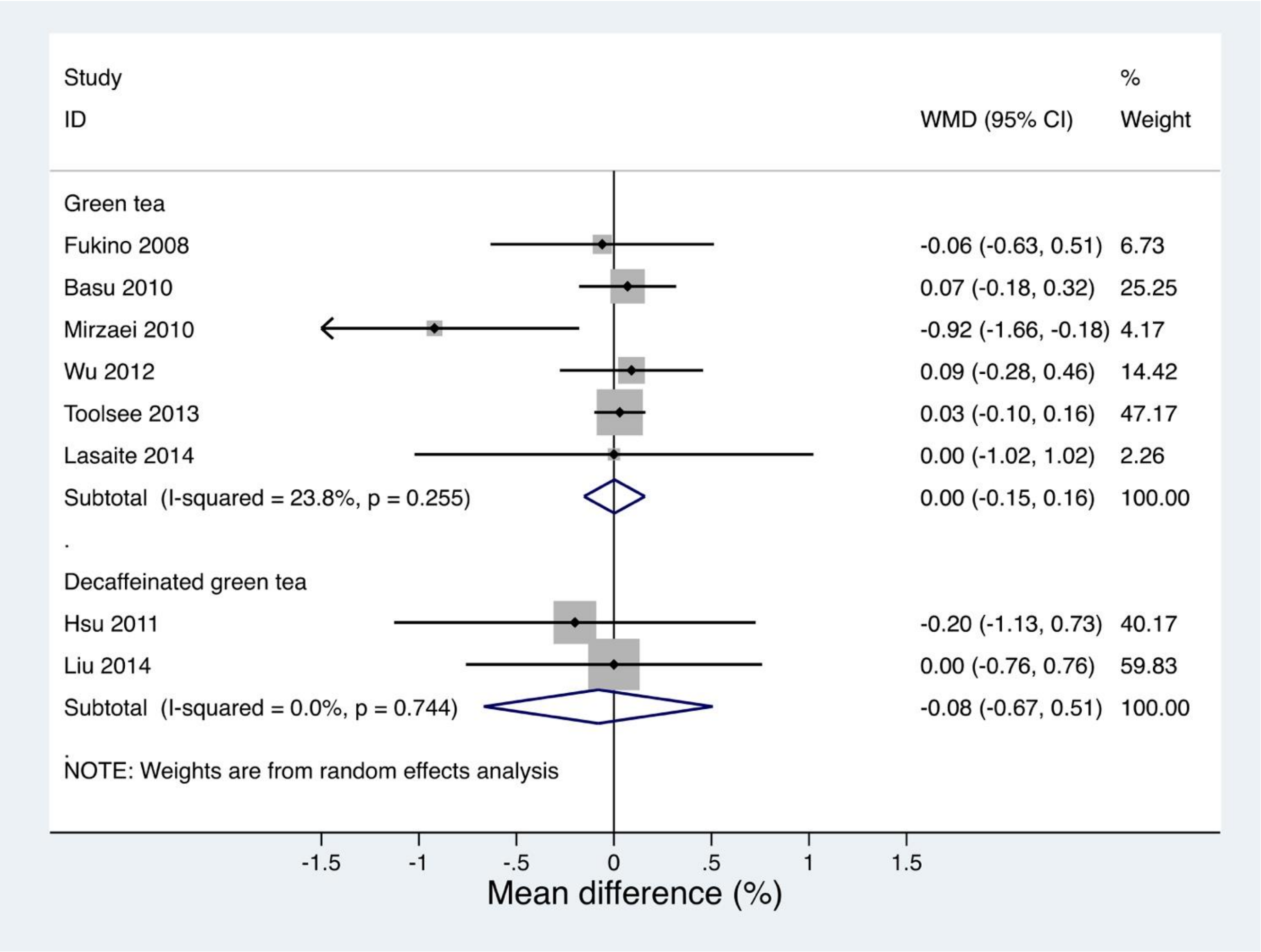

Supplemental Figure 5. Direct pairwise meta-analysis forest plot of effects on HbA1c vs. placebo/water. WMD, weighted mean difference

Supplementary Materials

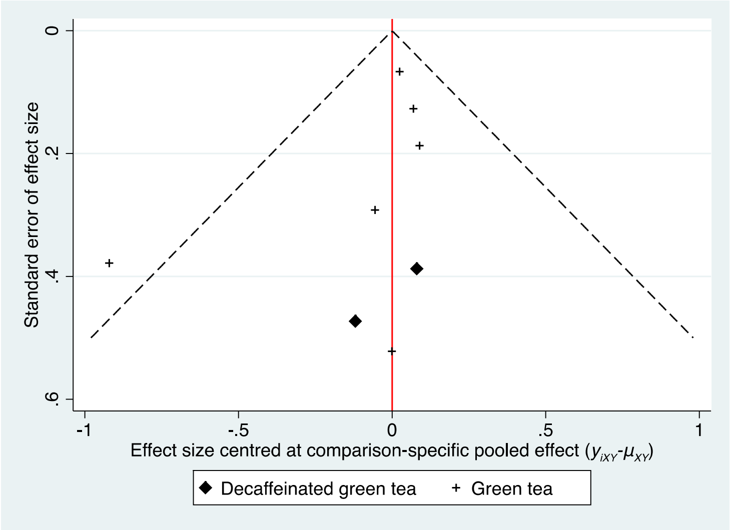

Supplemental Figure 6. Comparison-adjusted Funnel Plot of effects on HbA1c vs. placebo/water.

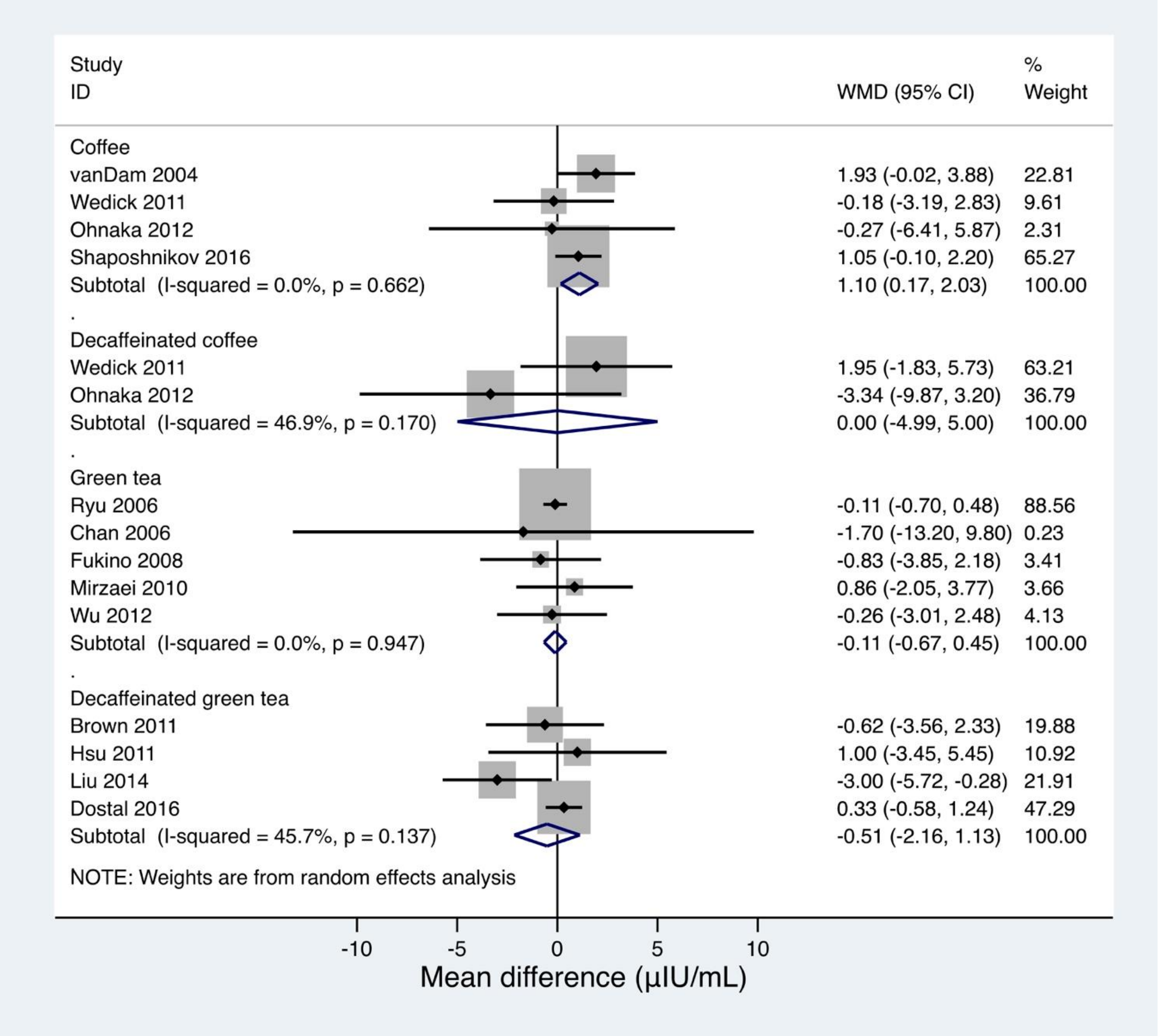

Supplemental Figure 7. Direct pairwise meta-analysis forest plot of effects on fasting blood insulin vs. placebo/water. WMD, weighted mean difference

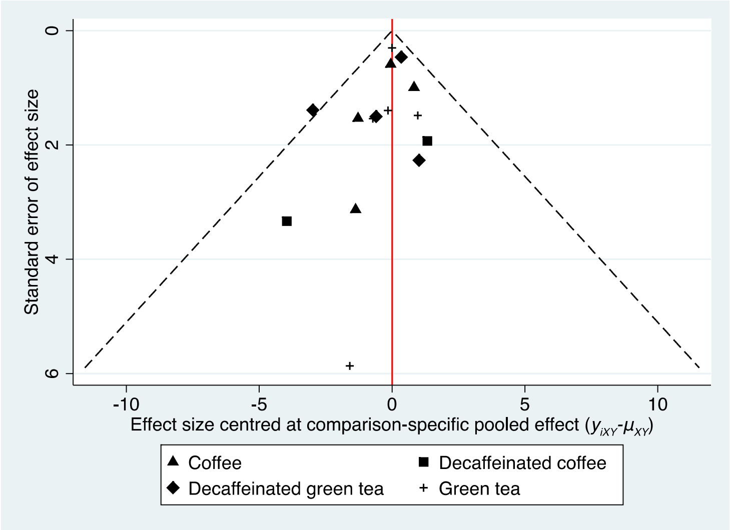

Supplemental Figure 8. Comparison-adjusted Funnel Plot of effects on fasting blood insulin vs. placebo/water.

Supplementary Materials

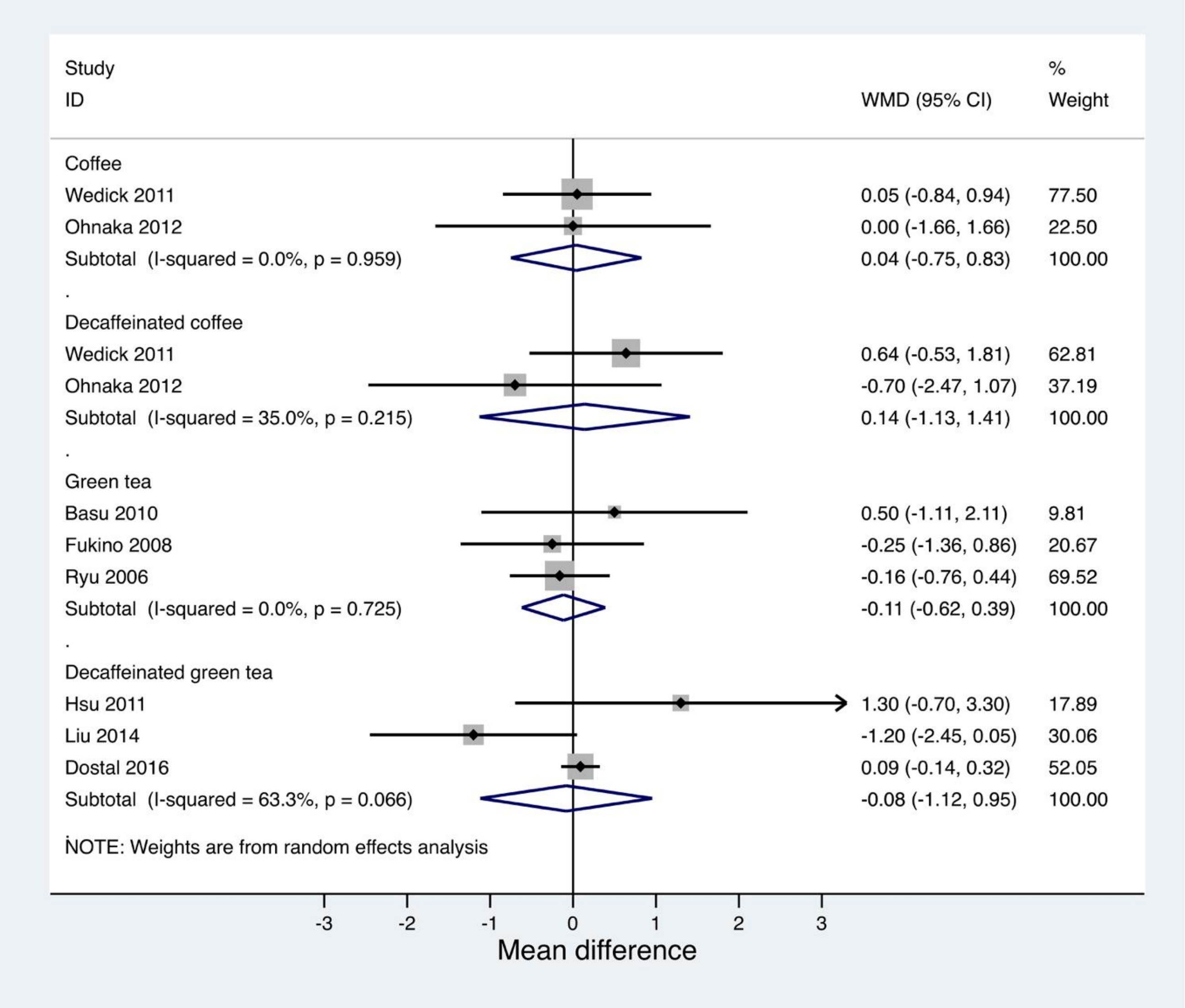

Supplemental Figure 9. Direct pairwise meta-analysis forest plot of effects on HOMA-IR vs. placebo/water. WMD, weighted mean difference

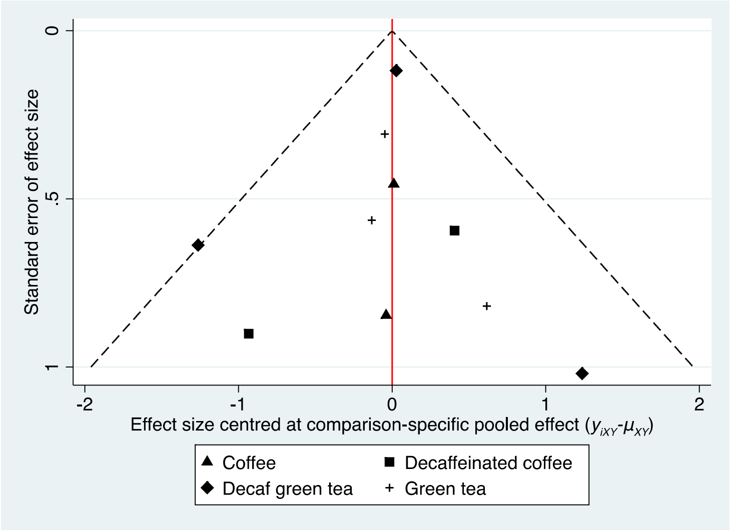

Supplemental Figure 10. Comparison-Adjusted Funnel Plot of effects on HOMA-IR vs. placebo/water.

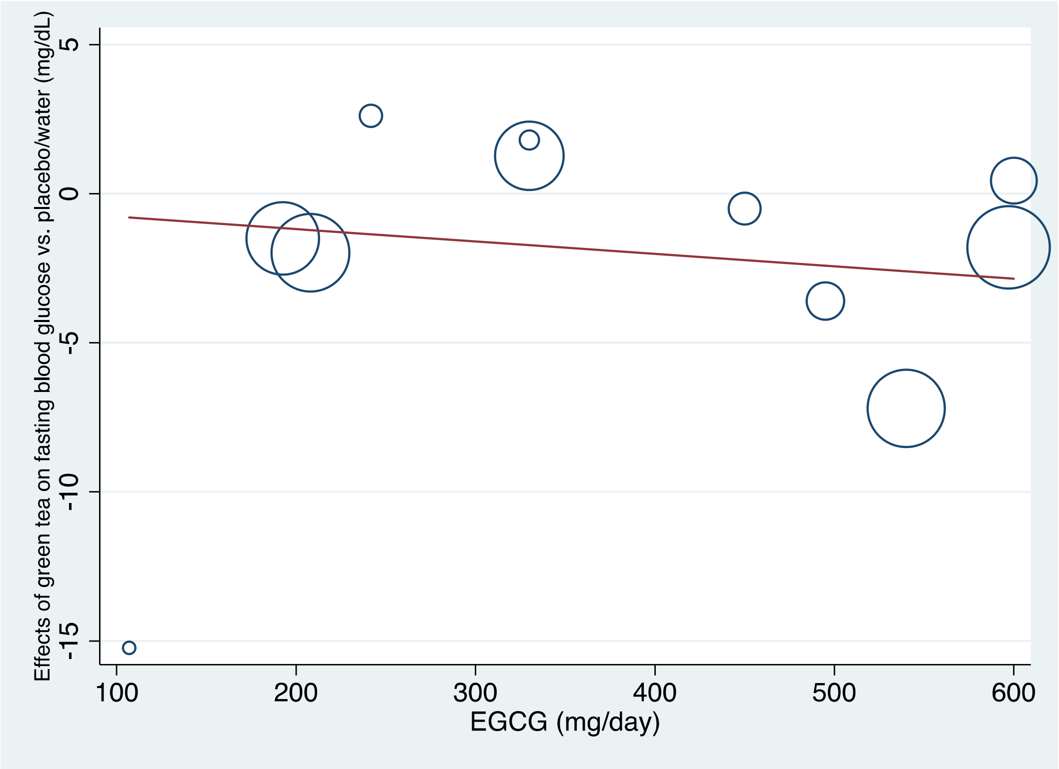

Supplemental Figure 11. Meta-regression graph between effects of daily EGCG dose contained in green tea on fasting blood glucose (β = 0.00, SE = 0.01, 95% CI -0.02 to 0.01, p = 0.54)
